# Current and future research on the association between gut microbiota and breast cancer

**DOI:** 10.3389/fmicb.2023.1272275

**Published:** 2023-10-30

**Authors:** Kuan Liu, Nan Jia, Hongyun Shi, Yuge Ran

**Affiliations:** Affiliated Hospital of Hebei University, Baoding, China

**Keywords:** gut microbiota, breast cancer, immune system homeostasis, estrogen, intracellular bacteria

## Abstract

Breast cancer (BC) is a prevalent malignancy. There exists a strong association between gut microbiota (GM) and the development of BC. The GM composition in individuals with BC significantly differs from that in their healthy counterparts. Furthermore, the distribution of GM varies significantly among individuals with different types of BC. The GM can impact BC through metabolite secretion, the gut-mammary axis, and other pathways. Modulating the GM can serve as a very promising potential therapeutic strategy in the treatment of BC. This article will summarize existing research, focusing on the relationship between intestinal microbiota and BC. At the same time, the project will also analyze the application value of intestinal microorganisms in BC intervention work, so as to provide a reference for the further development of BC prevention and treatment work.

## 1. Introduction

Breast cancer (BC) is a prevalent cancer affecting women and represents a significant global public health issue. According to the International Cancer Data Statistics (2020), there has been a substantial rise in the worldwide incidence of BC, with the number of new cases reaching a staggering 2.26 million. Furthermore, the incidence rate of BC is increasing at a rate of 0.5% each year, and the new cases are expected to grow to 3.20 million by 2050 (Ferlay et al., 2021). The gut microbiota (GM) is an intricate ecosystem containing trillions of intestinal microorganisms in a symbiotic relationship with the host. It plays a vital role in immune system homeostasis and metabolism in humans. Dysregulation of GM or changes in the relative abundance of specific gut bacterial species can precipitate the onset of a diverse range of pathological conditions. GM can modulate the production of chemokines and cytokines involved in immune regulation within the circulatory system. This alteration can form an immunological microenvironment supporting or inhibiting tumor growth (Hanahan, 2022). Tumor microbes have specific characteristics, and the composition of the temporal microbiome of solid tumors is closely related to the tumor type and its subtype (Livyatan et al., 2020). Imbalances in the GM have been associated with the development of a range of malignant tumors (Saud Hussein et al., 2021). The dysregulation of GM is one of the leading causes of BC, and it also affects the development of BC because of the gut-mammary axis and the dynamic evolution of metabolite-secreting glands (Alpuim Costa et al., 2021).

## 2. Modifications in the GM of BC patients

The gastrointestinal microbiota is the predominant microbial community within the organism. The human GM is characterized by a remarkably complex makeup comprising around 10×^13^ bacterial cells. Notably, the cellular content of gut microbes surpasses that of the human body by a factor of ten (Alpuim Costa et al., 2021). The GM serves a dual role in cancer development. On the one hand, some bacteria found in GM promote anti-tumor immunity against malignant tumors. Conversely, an asymmetry in genetic modification results in the formation of malignant neoplasms. The initial proposal of a correlation between genetic modifications and the risk of BC was put forward by Velicer et al. (2004).

The first illustration of the link between the GM and the clinical and biological characteristics of BC patients was provided by Goedert et al. (2015). It was noted that the composition of the GM was significantly altered in postmenopausal women with BC relative to healthy menopausal women and that the healthy menopausal group of women had a total estrogen and EM level of 22.36, a fecal microbiome of 91.2 species observable, and a Shannon index of 6.2. In contrast, the postmenopausal women with BC had a total estrogen and EM level of 45.40, 78.6 microbiomes observed in feces, and a Shannon index 6.0. The postmenopausal women with BC had a significantly lower diversity of intestinal bacteria, and the intestinal microbiota could impact the risk of BC in women (Goedert et al., 2015).

Numerous studies have linked the changes in the composition of GM with the clinical stage and tissue prognostic grading of BC patients. In a controlled study of the GM of BC survivors and healthy women, Caleça et al. (2023) noted that BC survivors had a higher abundance of GM communities, and that the relative abundance of the GM of BC survivors of 14 phylums, 29 orders, 25 orders, 64 families, 116 genera, and 74 species differed significantly from that of the healthy female population, and that BC survivors of the Bacteroidetes phylum, Firmicutes phylum, Verrucomicrobia phylum, *Clostridium* genus, *Shigella* genus, *Bifidobacterium* genus, *Akkermansia muciniphila, Clostridium perfringens, Escherichia coli, Bacteroides uniformis, Clostridium hathewayi*, and *Faecalibacterium prausnitzii* were significantly less abundant than in healthy females (Caleça et al., 2023). Aarnoutse et al. (2021) highlighted that the BC population exhibited the greatest prevalence of microbiota in the phylum characterized by thick-walled armies, followed by the phylum Anaplasma and Actinomycetes. The study suggests that neoadjuvant systemic therapy leads to a reduction in the abundance of the intestinal microbiota in patients diagnosed with BC. Furthermore, the stage of BC shows a negative correlation with the quantity of the *Veillonellaceae* and *Dialister*. Additionally, there is a clear association between the size of BC tumors and the decreased abundance of the *Veillonellaceae* (Aarnoutse et al., 2021).

Zhu et al. (2018) observed notable differences in the composition of GM among postmenopausal BC patients and postmenopausal healthy females. Specifically, they identified significant differences in 45 species of GM between these two groups. The GM of postmenopausal BC patients included *E. coli*, *Citrobacter*, *Acinetobacter radioresistens*, *Enterococcus gallinarum*, *Shewanella putrefaciens*, *Erwinia amylovora*, *Actinomyces sp. HPA0247*, *Salmonella enterica*, and *Fusobacterium nucleatum*. The species HPA0247, *Salmonella enterica*, and *Clostridium nucleatum* exhibited higher abundance levels, while microflora such as *Fusobacterium* and *Lactobacillus vaginalis* showed lower abundance levels (Zhu et al., 2018). Thu et al. (2023a) noted that the gut microbial α-diversity of BC patients was lower than that of healthy individuals and that there was uniqueness in the microbiota species of different BCs, with the BCER microbiota species being the most diverse and the BCTN tumor microbiota species the least diverse, and that the menopausal status was a typical driver of the variability of the GM in the BC population (Thu et al., 2023a). Bobin-Dubigeon et al. (2021) further explored the differences in GM composition between BC patients and healthy women. Compared to the healthy group, GM in the BC group demonstrated a significant loss of diversity, indicated by a decrease in the Shannon index, with the enrichment of the thick-walled bacteria phylum, *Clostridium XIVa cluster*, and *Clostridium IV cluster*. Meanwhile, the relative abundance of the phylum Anabaena, *Bifidobacterium sp.*, *Odoribacter sp.*, *Butyricimonas sp.* and *Coprococcus sp.* revealed a decreasing trend in BC patients (Bobin-Dubigeon et al., 2021).

In light of the discovery of GM changes in patients with BC, researchers have investigated the specific GM alterations for intervention in BC. Uzan-Yulzari et al. (2020) found that BC patients experience significant weight gain during adjuvant chemotherapy, which is an adverse prognostic factor for the quality of adjuvant chemotherapy for BC and decreases the survival of BC patients. Chemotherapy affects the body’s gut microbiome, and there may be an association between the composition of the gut microbiome and weight gain with adjuvant chemotherapy for BC. The pretreatment gut microbiome diversity and classification of women who gained weight after adjuvant chemotherapy differed markedly from that of women who did not gain weight, and patients with weight-gaining BC after chemotherapy had a higher relative abundance of *Dantoinaceae* and a more diverse pretreatment microbiome. The composition of the GM predicts weight gain after adjuvant chemotherapy, and the GM may contribute to adverse metabolic changes in the adjuvant chemotherapy population for BC (Uzan-Yulzari et al., 2020).

The studies mentioned above collectively emphasize the significant alterations observed in the GM of women diagnosed with BC, showing a strong relationship between these two variables. Microbiome characterization of different molecular subtypes of BC can be categorized into endocrine receptor positive (BERE), human epidermal growth factor receptor 2 positives (BEHR), estrogen, progesterone, and human epidermal growth factor receptor 2 triple-positive (BRTP), and HER2 receptor-deficient triple-negative (BRTN) type. Further, they are classified into four different molecular subtypes, namely luminal A (ER+/PR+ and Ki67 high), luminal B (ER+/PR+, Ki67 low or ER+/PR+/HE R2+), HER2 (HER2+) enriched, and basal types, as well as more significant microbial features in the tumor microenvironment of BCs as compared with that of healthy breast tissues. Microbial characteristics in BC may contribute to the distinct metabolic profiles observed in various molecular subtypes of BC. Banerjee et al. (2018) analyzed the different microbiological characteristics of various BC subtypes. A total of 17 viral features, 56 bacterial species, 21 fungal species, and 29 parasite features were observed to be more prominent in BC tissue compared to healthy breast tissue. Furthermore, it was found that the microbiome composition was distinct for each subtype, as shown in Table 1 (Banerjee et al., 2018). Nejman et al. (2020) noted that the microbiome of breast tumors was richer and more diverse than other tumors, with 16.4 species of bacteria being detected in samples from individual breast tumors, much higher than that of bacteria detected in different tumors.

**TABLE 1:** Characteristics of the microbiome for different subtypes of breast cancer.

Breast cancer types	Virus characteristics	Bacterial characteristics	Fungal characteristics	Parasitic signal characteristics
BERE		*Arcanobacterium* *Bifidobacterium* *Cardiobacterium* *Citrobacter* *Escherichia*	*Filobasidiella* *Mucor* *Trichophyton*	*Brugia* *Paragonimus*
BEHR	*Nodaviridae*	*Streptococcus*	*Epidermophyton* *Fonsecaea* *Pseudallescheria*	*Balamuthia*
BRTP	*Birnaviridae* *Hepeviridae*	*Bordetella* *Campylobacter* *Chlamydia* *Chlamydophila* *Legionella* *Pasteurella*	*Penicillium*	*Ancylostoma* *Angiostrongylus* *Echinococcus* *Sarcocystis* *Trichomonas* *Trichostrongylus*
BRTN		*Aerococcus* *Arcobacter* *Geobacillus* *Orientia* *Rothia*	*Alternaria* *Malassezia* *Piedraia* *Rhizomucor*	*Centrocestus* *Contracaecum* *Leishmania* *Necator* *Onchocerca* *Toxocara* *Trichinella* *Trichuris*

## 3. Mechanisms of action of GM affecting BC

### 3.1. Estrogen

**FIGURE 1:**
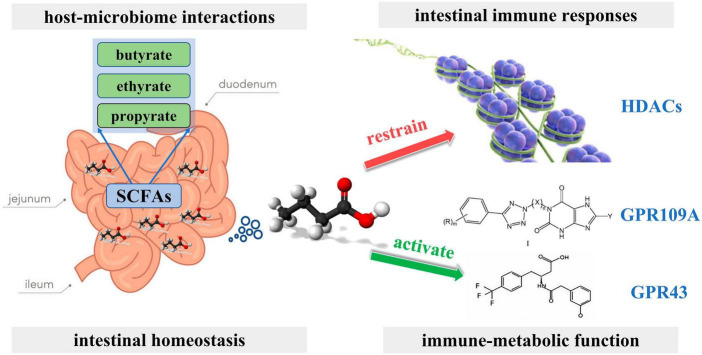
Impact of short-chain fatty acids on the gut microbiota.

Complicated etiology reveals beneath the BC. Although the exact biological events of BC are unclear, it is well established that endogenous estrogen is a significant risk factor in BC. Over 70% of BC patients have estrogen receptor-positive subtypes (Kwa et al., 2016). Estrogen metabolism is influenced by the variety of the intestinal microbiota, which is involved in controlling estrogen levels. One of the potential GM pathways influencing BC is the estrogen pathway. The steady decrease in C-27 cholesterol results in the production of estrogen, a steroid hormone. The primary forms of endogenous estrogen are estradiol, estrone, and estriol. Endogenous estrogens typically circulate in the bloodstream in free or protein-bound form to exert their biological effects. GM regulates the levels of circulating estrogens and influences the risk of hormone-driven malignancies through direct or indirect effects. The higher free estrogen levels in the gut can impact the relative plasma levels of estrogens and metabolites, increasing the risk of cancer progression (Figure 1; Kwa et al., 2016). Mani (2017) studied the relationship between endogenous estrogens, intestinal microbiota, and BC. They reported that ovaries, adrenal glands, adipose, and other tissues make up the total amount of estrogen in the body.

**FIGURE 2:**
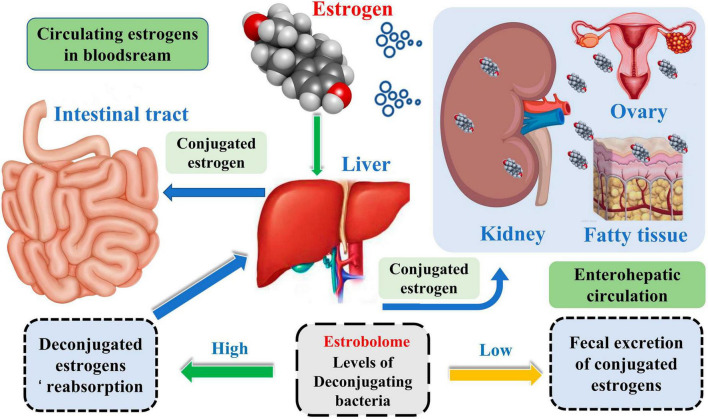
Enterohepatic circulation of estradiol group and estrogen.

**FIGURE 3:**
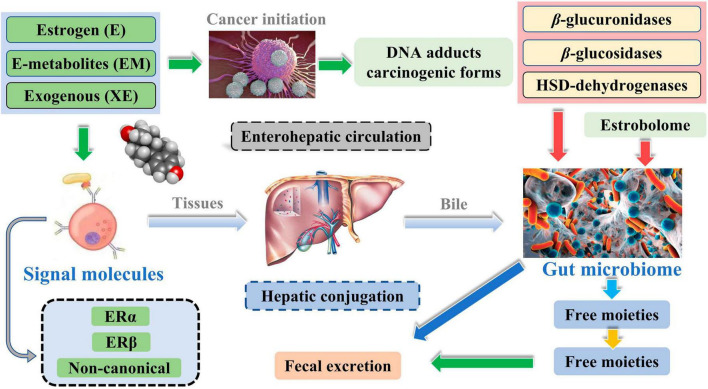
Potential impact of the estrobolome on estrogens.

Meanwhile, the liver is the leading site for metabolite formation, binding, and unbinding. Notably, microbial enzymes in the gut participate in the dissociation of estrogen, regulating the recirculation and total estrogen levels. Various circulatory metabolites impact the growth and development of estrogen receptor-positive BC (Figure 2; Mani, 2017). Parida and Sharma (2019) pointed out that endogenous estrogen is the main causative factor of BC in menopausal women. Before menopause, the ovary is the leading site of estrogen synthesis, and circulating estrogen acts in an endocrine manner on multiple targets. However, after menopause, estrogen production depends on the adipose tissue, bone, and the brain, serving in paracrine and endocrine manners. The microorganisms capable of metabolizing estrogen in the gastrointestinal tract were named “estrogenome (C-18 steroid hormones, estrogen E1, estrogen E2, estrogen E3).” The members of “estrogenome” regulate endogenous estrogen metabolism through β-glucuronidase and β-glucosidase activities via the hepatic-intestinal cycle to maintain estrogen homeostasis. The intestinal microbiota determines the excretion and recirculation of endogenous estrogens in postmenopausal women (Figure 3; Parida and Sharma, 2019).

### 3.2. Immune pathway

The intestinal immune system is an essential component of the human immune system, and the dysregulation of intestinal microbiota can impair the immune response, inducing various diseases, including extra-intestinal cancers. Rao et al. (2006) explored the role of *Helicobacter hepaticus*, an intestinal bacterial pathogen, in the induction of mammary carcinogenesis in C57BL/6 Apc Min/ + female mice. They found that *H. hepaticus* promoted mammary carcinogenesis through a tumor necrosis factor α-dependent (TNFα) mechanism, promoting epithelial tumorigenesis locally and at distant sites such as mammary glands (Rao et al., 2006). Immune dysregulation, which often originates from infection of the intestinal microbiota, is one of the causes of BC development. The mucosal surface of the intestines harbors a diverse population of microflora. During chronic infection by pathogenic gastrointestinal bacteria, the integrity of the intestinal epithelial barrier is compromised. This damage allows the bacteria to translocate from the intestine lumen to the underlying mucosal tissue (Rao et al., 2007).

Consequently, immune-inflammatory response cells become continuously activated, producing excess inflammatory cytokines. This dysregulation of immune activity involving persistent inflammation contributes to tumor formation (Rao et al., 2007). Loman et al. (2022) conducted a study investigating the correlation between BC and GM, utilizing samples obtained from mammary tumors in mice. It has been found that BCs generate pro-inflammatory cytokines, resulting in the compromise of intestinal barrier integrity. This phenomenon facilitates the translocation of intestinal bacteria and triggers systemic inflammation, leading to the manifestation of gastrointestinal and behavioral problems. In general, this procedure worsened the state of BC in mice (Loman et al., 2022).

Zhang et al. (2023) used an intestinal epithelial VDR knockout mouse model of BC to investigate the effects of vitamin D deficiency. The researchers discovered that a shortage in vitamin D resulted in an elevation of intestinal permeability, compromising the structural integrity of tight junctions within the intestines. Consequently, this phenomenon led to modifications in the composition of the intestinal microbiota, thereby facilitating the proliferation of BCs in terms of quantity and dimensions. Significantly, the findings of their study emphasized the interrelation among the gastrointestinal tract, mammary glands, and microbiome, indicating that preserving the intestinal barrier’s integrity might contribute to the onset of tumors outside the gastrointestinal system. Additional investigations can delve into preventative and therapeutic methodologies for extra-intestinal diseases like BC (Zhang et al., 2023).

Lakritz et al. (2015) reported that neutrophils are one of the primary immune factors in GM affecting BC development and progression. Neutrophils can induce genotoxic damage by producing reactive oxygen species, resulting in an initial mutagenic event of carcinogenesis.

In contrast, cytokines and chemokines secreted by tumor-associated neutrophils can shape the tumor microenvironment, contributing to tumor growth. Studies have demonstrated that GMs that face challenges can increase the activity of inflammatory cells in breast tissue. Conversely, the reduction of neutrophils throughout the body has been found to impede the establishment of breast tumors. Additionally, it has been demonstrated that the inflammatory response of GM has a role in the advancement of parenteral cancer by affecting neutrophil-mediated mechanisms (Lakritz et al., 2015).

### 3.3. Metabolite pathway

Intestinal microbial metabolites, such as amino acids, short-chain fatty acids, and hypocholesterolemic acid, are also involved in BC progression. The metabolic pathway exhibits immunoregulatory properties in BC. The GM can convert tryptophan found in the food into several indole derivatives. These derivatives serve as ligands for the aromatic hydrocarbon receptor pathway, an immunomodulator of the gastrointestinal tract. The upregulation of the aromatic hydrocarbon receptor can amplify tryptophan metabolism-inducing BC cells, leading to apoptosis (Bekki et al., 2015). Cadaverine, a product of bacterial decarboxylation of lysine and arginine, contributes to lower gut pH, affecting bacterial growth. Relative to healthy individuals, BC individuals had a lower abundance of Enterobacteriaceae CadA as well as *E. coli*, *Enterobacter cloacae*, and Hafnia LDC DNA, and the microbiota as mentioned above were primarily responsible for bacterial cadaverine production, resulting in significantly lower levels of cadaverine in BC individuals than in healthy individuals. Cadaverine can induce mesenchyme transformation into epithelial cells via the TAAR receptor *in vitro* and *in vivo*. It exhibits a reduction in tumor infiltration into peripheral organs and effectively manages the occurrence of distant metastasis.

Furthermore, the compound known as cadaverine has demonstrated significant efficacy in suppressing the proliferation, migration, invasion, and stem cell characteristics of BC cells. It is worth noting that during the initial phase of BC, there is a notable decrease in the synthesis of cadaverine within the human body. A correlation exists between diminished levels of cadaverine production and a reduction in the diversity of gut bacteria populations (Kovács T. et al., 2019).

**FIGURE 4:**
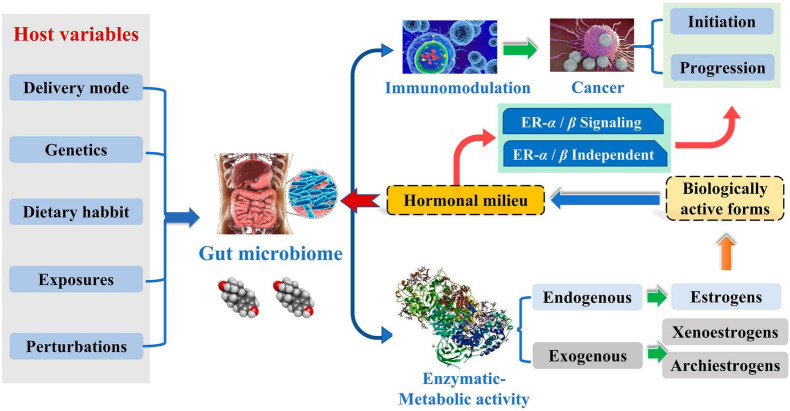
Potential association of intestinal estralol group with breast cancer risk.

Short-chain fatty acids (SCFAs), Including butyric, propionic, and acetic acids, are the most studied gut microbial metabolites. SCFAs are produced in the small intestine and are important microbial messengers of the immune system. SCFAs are involved in complex host-microbiome interactions and intestinal immune responses. They exert anti-inflammatory effects and enhance microbial pathogen tolerance by inhibiting histone deacetylase and activating anti-inflammatory cytokines produced by GPR109A and GPR43. SCFAs play a positive influence on intestinal homeostasis as well as on the immune-metabolic function of the body (Figure 4; Aarnoutse et al., 2022). The study by Yoon et al. (2019) evaluated the association between the physiological intestinal absorption of BC patients and their intestinal bacteria composition. According to the research, the Ruminalococcaceae family has been found to have a preference for the generation of butyric acid, which serves as a crucial microbial regulator of immunological function inside the intestinal mucosa. Consequently, this family can serve as a sign of a well-functioning anaerobic intestinal microbiota.

Moreover, the abundance of butyrate-producing bacteria decreases in the GM of BC patients, significantly correlating with mucosal destruction and increased mucosal inflammation in BC (Yoon et al., 2019). Butyrate is transported into cells by the SMCTI transporters and distributed throughout the body by other transporter proteins, resulting in a systemic impact. The excessive buildup of intracellular butyrate can potentially contribute to the onset and progression of malignant diseases. In the context of BC, it has been observed that butyrate can restrict the proliferation of BC cells. This effect is achieved through its role as an inhibitor of histone deacetylase (HDACi), which stimulates the production of p21. This kinase inhibitor operates inside the cytosol and depends on cytosolic proteins (Mirzaei et al., 2021).

A study noted significant changes in the composition and symbiosis of the intestinal microbiota in pre-menopausal BC patients relative to healthy pre-menopausal women. The BC patients showed a substantial reduction in the abundance of SCFA-producing bacteria and related enzymes. Compared with normal breast cells, propionate and butyrate reduce the activity of BC cells in a dose-dependent manner. This again highlights that SCFAs can help slow down the progression of BC, mainly through regulating immune function and inflammatory factors, such as T-cell and histone deacetylase activities (He et al., 2021).

Bile acids are soluble derivatives of cholesterol produced in the liver that are converted by gut microbes to form a variety of metabolites. In the gut, a portion of bile acids are converted by gut bacteria into secondary bile acids, which are retained in the circulation and mediate receptor-mediated effects on cancer cells through the extracellular signal-regulated kinases ERK and PKC, which induce DNA binding and trans-activation of activator protein 1 (AP-1). Secondary bile acids were recognized as a liver and colon cancer risk factor. Lithocholic acid, a type of secondary bile acid, was shown to inhibit the proliferation and invasion of BC cells through TGR5 (Mikó et al., 2018). In the early stages of BC, the diversity of the gut microbiome decreases, along with the production of inhibitory bacterial metabolites such as *lithocholic acid*. The expression of key components in the secondary bile acid-induced cytostatic pathway (iNOS and 4HNE) decreases with the progress of BC. Lipid peroxidation levels in tumors negatively correlate with mitotic index. In triple-negative cases, overexpression of iNOS, nNOS, CAR, KEAP1, NOX4, and TGR5 or downregulation of NRF2 was related to improved BC patient survival. Secondary bile acids cause oxidative stress, which slows BC cell proliferation (Kovács P. et al., 2019). Sodium bile deoxycholate (SBDC), synthesized by intestinal bacteria, facilitates the processing of host dietary fats. Also, SBDC was shown to reduce the crude apoptotic neuro-adenocarcinoma levels and promote the growth of tumor cells by upregulating Flk-1 (Krishnamurthy et al., 2008; Franco, 2022).

### 3.4. Enteric bacteria

Bacteria are an essential component of tumors and are found in the cytoplasm of immune and tumor cells. Various intracellular bacteria in BC tissues have been confirmed to play a vital role in the metastasis of BC (Fu et al., 2022). Parida et al. (2021) reported that *Bacteroides fragilis* is a common colonizing bacterium that asymptomatically lives in individuals, and its virulence is attributed to zinc metalloproteinase toxins. *B. fragilis* can influence the structure of the gut microbial community based on its secretion products and is a significant factor in diarrhea, inflammatory bowel disease, and carcinomatous transformation of the intestinal mucosa. *B. fragilis* toxins can promote tumor growth and metastasis in BC through intestinal and intraductal colonization via the β-catenin and Notch1 pathways (Parida et al., 2021). Alterations in intestinal microbiota play a key role in tumor development and suppression (Kondegowda et al., 2011). Microbial peptides from intracellular bacteria can remodel the tumor microenvironment (TIME) by providing targets for CTL and CD4+ T cells under the presentation of MHC class I or II molecules on the surface of tumor cells (Terrisse et al., 2023). For instance, *H. hepaticus* is a pathogen found in mice’s liver and intestines. In mammary tumor-prone mice, *H. hepaticus intestinalis* was shown to promote mammary and intestinal tumors. Furthermore, the commensal bacteria in the tract metastasize to distant organs and promote cancer progression. *H. hepaticus*-induced dysbiosis of the intestinal microbiota promoted the systemic spread of bacteria. Intestinal bacterial subpopulations can move from the gut to the breast via the lymph nodes and establish a pro-inflammatory microenvironment (Deng et al., 2022).

## 4. GM and BC treatment

As the relationship between GM and BC becomes more explicit, researchers have been increasingly exploring the role of GM in BC therapeutic approaches. Manipulation of GM is the most promising future technique for achieving treatment efficacy in BC or discovering therapeutic biomarkers (Plottel and Blaser, 2011; Mikó et al., 2019), and GM is causal in modulating host immunity and the effectiveness of chemotherapy and immunotherapy (Di Modica et al., 2021). A potential association exists between GM and the efficacy of anti-malignant medications and immunotherapy in individuals diagnosed with solid tumors. Furthermore, the GM’s ability to modulate immune checkpoint inhibitors may give rise to novel therapeutic prospects for managing solid tumors (Llamosas-Falcón et al., 2022; Vitorino et al., 2022).

The currently available body of research has demonstrated the potential utility of GM regulation in treating BC. Significantly, probiotics can modulate immune responses and impact the GM. Hence, probiotics possess the potential to actively manipulate the GM and successfully regulate the progression of BC (Thu et al., 2023b). Xue et al. (2018) reported that GM can drive estrogen metabolism, and fucoidan as a GM regulator may prevent BC. Mice with BC were fed with fucoidan and showed improvement in the diversity and composition of GM, promoting intestinal barriers that help prevent BC (Xue et al., 2018). Bawaneh et al. (2022) reported the genetic modification of female mice using high-fat diet-derived (HFD)-FMT, which increased the risk of BC in females. *A. muciniphila* is a gut microbe that promotes the Dox response, and *Encephalitozoon intestinalis caecimuris* is a microbe that is associated with reduced therapeutic efficacy. A high-fat diet will have an impact on both of these gut microbes has an impact on both of these gut microbes, altering the GM. In addition, the modulatory effects of antibiotics, HFD-FMT, or lipopolysaccharide on GM affected the response of mice to TNBC chemotherapy, lung metastasis, and intestinal inflammation. Based on these observations, the authors concluded that genetic modification is a plastic, adaptable, and targetable BC risk factor (Bawaneh et al., 2022). Gut microbes can convert plant lignans into enterolignans, enterodiols, and enterolactones. As GM modulators, lignans can be used to improve estrogen regulation and inhibit/delay the growth of BC (Saarinen et al., 2007). Goldfinch isoflavone was shown to regulate GM in mice, which prolonged mammary tumors’ latency period, reducing their development (Paul et al., 2017).

Ji et al. (2023) developed a BC diagnostic model based on the intriguing relationship between GM and BC. They pointed out that with the advancement of genome sequencing, the detection of the gut microbiome can be realized *in vivo* to find changes in GM, leading to the development of tumors. 16S rRNA sequencing was performed to analyze the change in GM during breast tumorigenesis, revealing key GM biomarkers that can be used as a criterion for predicting the occurrence of tumors (Ji et al., 2023).

## 5. Summary and outlook

In conclusion, a preliminary connection has been found between GM and BC, which holds the potential for informing the development of preventative and treatment initiatives for BC. Nevertheless, most current research heavily depends on identifying fecal microbiota, with a specific emphasis on the association between GM and BC. However, only a limited number of studies have investigated the cause-and-effect relationship to explore the fundamental mechanisms that link these two factors.

Therefore, to maximize the utilization of GM in BC diagnosis, treatment, and prognosis, it is imperative to enhance forthcoming investigations about the mechanisms that establish a connection between GM and BC. Through employing several methodologies, investigating this association can yield an innovative clinical strategy for treating BC.

## Author contributions

KL: Visualization, Writing – original draft. NJ: Visualization, Writing – original draft. HS: Conceptualization, Writing – review and editing. YR: Writing – review and editing.
